# Meetings of Societies

**Published:** 1914-12

**Authors:** 


### MEETINGS OF SOCIETIES.

### Bristol Medico-Chirurgical Society.

*Annual Meeting, October 14th, 1914.*

After a vote of thanks to the retiring President, Dr. P. Watson-Williams, had been passed, Dr W. H. C. Newnham took the chair, and gave an introductory address (*see* p. 257). Later a cordial vote of thanks was given to Dr. Newnham for his able and interesting address.

The Acting Honorary Secretary, Dr. J. M. Fortescue-Brickdale, read the following annual report :—

During the year eighteen new members had been elected and fourteen had resigned. The Society had to regret the loss of one member by death. There was now a total of four honorary and 174 ordinary members.

The balance from the previous year was £98 2s. 3d., and on this year there was a balance of £14 12s. 6d.

The average attendance at meetings had been 35.

The Hon. Librarian, Mr. C. K. Rudge, then read the following report :—

The Library of the Bristol Medico-Chirurgical Society now contains 12,867 volumes. During the year 616 volumes have been added, of which 86 have been received for review in the *Journal* of the Society, and the remainder by gift or exchange. The Library is in regular receipt of 191 periodicals, 28 of which are purchased and 163 received in exchange.

During the year 754 issues of books were made to 93 members, and 28 volumes had been obtained from Lewis's Library.

The Bristol Medical Library, to which members have access, now contains 23,576 volumes, and 253 British and foreign periodicals. The number of volumes mentioned was made up in the following manner :—

BOOKS.

| | |
|---|---|
| Bristol Medico-Chirurgical Society .. | 12,867 |
| University of Bristol .. .. .. | 8,946* |
| Bristol Royal Infirmary .. .. | 1,189 |
| Bristol General Hospital .. .. | 574 |
| Total .. .. | 23,576 |

* An increase of 164.

CURRENT PERIODICALS.

| | |
|---|---|
| Medico-Chirurgical Society .. .. | 191 |
| University of Bristol .. .. .. | 61 |
| Total .. .. | 252 |

The following are the officers of the Society for the Session 1914–15 : President—W. H. C. Newnham, M.A., M.B. Cantab. ; President-Elect—G. Parker, M.A., M.D. Cantab. Committee (*ex-officio*)—P. Watson-Williams, M.D. Lond. (Past President and Editor of *Journal*) ; C. K. Rudge, M.R.C.S. (Hon. Librarian) ; J. M. Fortescue-Brickdale, M.A., M.D. Oxon. (Editorial Secretary) ; A. J. Wright, M.B. Lond. (Hon. Secretary). (*Elected*)—R. Shingleton Smith, M.D. Lond. ; G. Munro Smith, M.D. Bristol ; I. Walker Hall, M.D. Vict. ; J. Lacy Firth, M.D. Lond. ; J. O. Symes, M.D. Lond. ; L. E. V. Every-Clayton, M.D. Lond. ; F. H. Edgeworth, M.D. Cantab. ; H. S. Ballance, M.D. Lond. Hon. Secretary—A. J. Wright, M.B. Lond. Acting Hon. Secretaries—J. A. Nixon, B.A., M.B.

Cantab. (*Financial*) ; J. M. Fortescue-Brickdale, M.A., M.D. Oxon. (*General*). Library Committee—C. K. Rudge, M.R.C.S. ; G. Munro Smith, M.D. Bristol ; W. A. Smith, M.A., M.B. Oxon.

**RECEIPT AND EXPENDITURE ACCOUNT OF THE BRISTOL MEDICO-CHIRURGICAL SOCIETY, SESSION 1913-14.**

| | £ | s. | d. | | £ | s. | d. |
|---|---|---|---|---|---|---|---|
| Balance from 1912–13 .. | 98 | 2 | 4 | Honorarium (Farr) .. | 10 | 0 | 0 |
| Members' Subscriptions | 156 | 9 | 0 | Postage .. .. .. .. .. | 1 | 0 | 0 |
| Interest on Deposit .. | 0 | 19 | 6 | Library Grant .. .. | 30 | 0 | 0 |
| | | | | Telephone .. .. .. | 6 | 10 | 0 |
| | | | | Lanterns .. .. .. | 1 | 1 | 0 |
| | | | | Postage .. .. .. .. .. | 3 | 0 | 0 |
| | | | | Fortt (refreshments) .. | 1 | 19 | 0 |
| | | | | Cheque Book .. .. | 0 | 2 | 6 |
| | | | | Meeting Expenses 11/2/14 | 0 | 17 | 6 |
| | | | | Lantern .. .. .. | 1 | 1 | 0 |
| | | | | Lantern .. .. .. | 0 | 15 | 3 |
| | | | | Meeting Expenses .. | 0 | 10 | 0 |
| | | | | University Marshal .. | 1 | 11 | 6 |
| | | | | Library Grant .. .. | 50 | 0 | 0 |
| | | | | Trübner .. .. .. | 0 | 19 | 7 |
| | | | | *Journal* Grants .. .. | 75 | 0 | 0 |
| | | | | Arrowsmith .. .. .. | 2 | 9 | 6 |
| | | | | Auditor .. .. .. .. .. | 0 | 10 | 6 |
| | | | | Rent to University .. | 52 | 10 | 0 |
| | | | | Petty Cash .. .. .. | 1 | 1 | 0 |
| | | | | Balance in Bank .. .. | 12 | 10 | 6 |
| | | | | Balance in hands of Acting Secretary .. .. | 2 | 2 | 0 |
| | £255 | 10 | 10 | | £255 | 10 | 10 |

*November* 11*th*, 1914.

Dr. W. H. C. Newnham, President, in the Chair.

Dr. Irving, in the absence of Dr. P. Watson-Williams, showed a case of **Nasal Pansinusitis** in which the intersinus frontal septum had been deliberately broken down pernasally so as to assure a thorough flush through. The maxillary and sphenoidal sinuses had also been opened intranasally.—Dr. Every-Clayton spoke on the apparent advantages from breaking down the septum, and on two successful cases of which he had knowledge in which the patients had derived great benefit from the intranasal operation for frontal sinusitis.

Dr. G. Munro Smith read **Notes on some of the Bristol Resurrectionists.** Drs. Shingleton Smith and Parker discussed the paper.

Dr. O. C. M. Davis read a paper on **The British Pharmacopœia of 1914** (Page 292). Drs. Neild and Fortescue-Brickdale discussed the paper.

J. Lacy Firth.
J. M. Fortescue-Brickdale,
*Acting Hon. Sec.*

